# *In vitro* model for the assessment of human immune responses to subunit RSV vaccines

**DOI:** 10.1371/journal.pone.0229660

**Published:** 2020-03-19

**Authors:** Tatiana Chirkova, Binh Ha, Bassam H. Rimawi, Antonius G. P. Oomens, Tina V. Hartert, Larry J. Anderson

**Affiliations:** 1 Department of Pediatrics, Division of Infectious Diseases, Emory University School of Medicine, Emory University, Atlanta, Georgia, United States of America; 2 Maternal-Fetal Medicine, WakeMed Health & Hospitals, Raleigh, North Carolina, United States of America; 3 Department of Veterinary Pathobiology, Center for Veterinary Health Sciences Oklahoma State University, Stillwater, Oklahoma, United States of America; 4 Department of Medicine, Division of Allergy, Pulmonary & Critical Care Medicine, Vanderbilt University Medical Center, Vanderbilt University, Nashville, Tennessee, United States of America; University of Georgia, UNITED STATES

## Abstract

Respiratory syncytial virus (RSV) is the single most important cause of serious lower respiratory tract disease in infants and young children worldwide and a high priority for vaccine development. Despite over 50 years of research, however, no vaccine is yet available. One block to vaccine development is an incomplete understanding of the aberrant memory response to the formalin-inactivated RSV vaccine (FI-RSV) given to children in the 1960s. This vaccine caused enhanced respiratory disease (ERD) with later natural RSV infection. Concern that any non-live virus vaccine may also cause ERD has blocked development of subunit vaccines for young children. A number of animal FI-RSV studies suggest various immune mechanisms behind ERD. However, other than limited data from the original FI-RSV trial, there is no information on the human ERD-associated responses. An *in vitro* model with human blood specimens may shed light on the immune memory responses likely responsible for ERD. Memory T cell responses to an antigen are guided by the innate responses, particularly dendritic cells that present an antigen in conjunction with co-stimulatory molecules and cytokine signaling. Our *in vitro* model involves human monocyte derived dendritic cells (moDC) and allogenic T cell cultures to assess innate responses that direct T cell responses. Using this model, we evaluated human responses to live RSV, FI-RSV, and subunit RSV G vaccines (G-containing virus-like particles, G-VLP). Similar to findings in animal studies, FI-RSV induced prominent Th2/Th17-biased responses with deficient type-1 responses compared to live virus. Responses to G-VLPs were similar to live virus, i.e. biased towards a Th1 and not a Th2/Th17. Also mutating CX3C motif in G gave a more pronounced moDC responses associated with type-1 T cell responses. This *in vitro* model identifies human immune responses likely associated with ERD and provides another pre-clinical tool to assess the safety of RSV vaccines.

## Introduction

Respiratory syncytial virus (RSV) is a leading cause of acute, severe lower respiratory tract disease in young children with an estimated 3.2 million cases of severe lower respiratory tract illness and 48,000–74,500 in-hospital deaths in children younger than 5 years of age [1–3]. It is also estimated to cause 14,000 deaths each year in adults in the United States and associated with later development of reactive airway disease or asthma [4, 5]. Its substantial disease burden has made RSV a priority for vaccine development for over 50 years but, unfortunately, no vaccine is yet available. Four target populations, i.e. young children (≤6 months of age), older children (6 months to 24 months of age), pregnant women, and elderly adults (≥65 yrs. of age) [3, 6], have been the focus of vaccine development and each has different concerns for vaccine safety and efficacy. The fact that natural infection provides limited protection from subsequent infection and disease highlights one challenge to achieving an RSV vaccine–inducing protective immunity. Other challenges include: the young age of peak risk of disease, i.e. children 2–4 months of age; lack of reliable correlates of protection; inability of pre-clinical animal studies to predict how a vaccine will do in the human target population; and the enhanced respiratory disease seen in young, RSV-infected children who earlier received a formalin-inactivated RSV with alum adjuvant (FI-RSV) vaccine. It is likely that the FI-RSV vaccine induced an aberrant memory response in young, likely RSV naïve, children that caused enhanced respiratory disease (ERD) with subsequent natural RSV infection [7–10]. Young FI-RSV vaccinated children, but not older children who likely were RSV primed, had a high rate of hospitalization and two died with later RSV infection. This experience raised the concern that any non-live virus vaccine may cause ERD in young children and limited vaccine development for young children to live virus vaccines. Unfortunately, no live virus vaccine has yet been sufficiently safe and immunogenic to move to licensure. In older children and adults who are RSV primed, ERD is not a concern and, since live RSV vaccines are poorly immunogenic in RSV primed persons, non-live subunit vaccines are under development for these target populations. The many failed attempts to develop an RSV vaccine suggest new strategies may be needed to finally achieve success [3].

Though there is presently no path to use a non-live subunit vaccine in young children, there are potential advantages to subunit vaccines. A subunit vaccine does not have the risk of disease associated with virus replication that a live virus vaccine does, and provides options for enhancing protective immune responses, e.g. through antigen design or adjuvants, that are not available in a live RSV vaccine. Since an aberrant memory T cell responses is likely important to FI-RSV induced ERD [11, 12], a way to study the human memory T cell responses would help the evaluation of subunit vaccines.

At present a vaccine’s risk of predisposing to ERD is assessed in animal studies by comparing responses to FI-RSV, benchmark for ERD risk, and live virus, benchmark for a safe response. This evaluation follow immune and inflammation responses after the viral challenge of FI-RSV and live virus immunized mice. In these studies, a predominant Th2 and Th17 biased responses are associated with FI-RSV vaccination, and predominant type-1 biased responses with live virus infection [13–18]. However, other than limited data from the original FI-RSV trial, there is no information on the human ERD-associated responses. Since a FI-RSV vaccine cannot be studied in humans, an *in vitro* model of the human immune response is, at present, the only way to assess the memory response a vaccines is likely to induce. Our *in vitro* model takes advantage of the central role that measurable dendritic cell responses play in guiding development of memory T cell responses. Dendritic cells that present an antigen in conjunction with co-stimulatory molecules and cytokine signaling are associated with induction of Th1, Th2 and Th17 responses. With our *in vitro* model we compared FI-RSV stimulation to live RSV stimulation, and found FI-RSV inducing human responses similar to the ones defined as ERD-risk associated in animal models. FI-RSV, in contrast to live RSV strains, induces DC responses associated with the differentiation of Th2 and Th17 T cells, while live RSV induced DC responses associated with more type-1 biased responses. Thus, this model provides an *in vitro* pre-clinical method to evaluate human ERD-risk responses to a vaccine.

We then, chose to test this in vitro model with candidate vaccines, we are developing in our laboratory. Although current RSV subunit vaccines that are under development primarily focus on the F protein which induces high levels of cross reactive neutralizing antibodies and protection in animals, G protein vaccines also have potential. G protein, because of its role in disease pathogenesis in animal studies and its impact in binding to cells in human infection, makes a promising vaccine component and valuable addition to an F protein vaccine. Animal studies show that G induces host responses that contribute to many features of RSV disease including increased inflammation, mucus, and type 2 cytokines in the lung and increased airway resistance [19–21]. G’s role in disease is, at least in part, mediated through its CX3C chemokine motif binding to the corresponding fractalkine receptor, CX3CR1 [22]. Blocking the central conserved domain of G, which encompasses CX3C motif, with monoclonal antibody or mutating the CX3C motif decreased disease in mice both with primary RSV infection and RSV challenge after FI-RSV vaccination [19–21, 23–25]. Additionally, a G peptide vaccine given one day after administration of an FI-RSV vaccine to mice, blocked much of the ERD lung inflammatory response with later RSV challenge [26]. Overall, these data suggest that G protein could be an important addition to a subunit RSV vaccine and, thus, was chosen as a vaccine candidate for our in vitro model of the likely human response to a vaccine.

In this report, we describe *in vitro* model of ERD-risk human immune responses by evaluating the monocyte-derived dendritic cell (moDC) directed T cell responses to FI-RSV, live RSV and RSV G-VLPs. We found human ERD-risk responses (to FI-RSV) and non-ERD responses (to live RSV) to be consistent with outcomes predicted in animal FI-RSV studies. The G-VLP vaccines evaluated in this *in vitro* model induced responses similar to live RSV and not FI-RSV, suggesting that addition of G and/or G with a mutated CX3C motif is worth considering as a component of a subunit vaccine. Additionally, our *in vitro* model of human immune responses provides another pre-clinical tool to assess the safety of RSV vaccines.

## Materials and methods

### Viruses and VLP

Two RSV A2 derived strains, wild-type and CX3C-mutant, were generated with a reverse genetic system as previously described [27]. The wild-type strain (RSV-wt) has an intact CX3C motif (182CWAIC186) in G protein and is parent to the mutant strain (RSV-CX4C) which has an alanine A186 insertion in the CX3C motif (182CWAIAC187). The mutant strain was prepared as described [28]. Viral stocks were propagated in HEp-2 cells (ATCC, Manassas, VA), purified by pelleting through 20% sucrose cushion at 18,000×g for 2 h, re-suspended in Minimal Essential Media (MEM, Gibco, Thermo Fisher Scientific, Waltham, MA) without FBS and stored at -80°C. Viral genomes were verified by harvesting viral RNA from infected cells, amplification by RT-PCR, and bulk sequence analysis.

Formalin-inactivated RSV (FI-RSV) was prepared from the wild-type A2 RSV as described [10]. Briefly, freshly propagated viral stock was inactivated by adding formalin at 1:4000 final dilution, incubating for 72 h at 37°C, then purified and concentrated by pelleting through 20% sucrose cushion at 18,000×g for 2 h and re-suspension in 1/25 of the original volume in MEM without FBS. Concentrated virus was then alum-precipitated by incubating overnight at room temperature with 4 mg/ml of aluminum hydroxide. The precipitated virus was then pelleted by centrifugation at 500×g for 15 min at 4°C, and re-suspended in MEM at 1/100 of the original volume.

Mock and FI-Mock were prepared from the un-infected HEp-2 cells in the same manner as live or FI-inactivated RSV. Live and FI-RSV in each experiment were prepared from the same batch of freshly propagated virus.

The wild-type G and CX4C mutant G VLPs (VLP-G(wt) and VLP-G(CX4C) respectively) were generated as described for Gag-Env VLP [29]. Briefly, codon optimized HIV gag and RSV A2 G protein genes were co-expressed in 293F cells for 48–72 h followed by low-speed centrifugation, filtration through 0.45 μm filter, pelleting through 20% sucrose cushion, purification on linear 20–60% sucrose gradients, and pelleting the VLP containing fractions for 2 h at 12,000×g. Purified VLP were re-suspended in PBS. The G content of the re-suspended VLP was determined by Western blot and ELISA (S1A and S1C Fig) standardized to purified G protein (BEI Resources, Manassas, VA). The expression of G on the surface of VLP was confirmed by electron microscopy with immune-gold staining (S1B Fig).

### Monocyte-derived dendritic cells (moDC) and allogenic naïve CD4 T cell isolation

Peripheral blood from healthy adult donors and cord blood was collected under an IRB-approved protocol and provided to the laboratory delinked from personal identifiers. Peripheral blood (PBMC) and cord blood mononuclear cells (CBMC) were isolated by centrifugation on a gradient of Ficoll-Paque PREMIUM (GE Healthcare, Uppsala, Sweden). MoDC were prepared as described [30]. Briefly, freshly-isolated PBMC/CBMC were plated in Monocyte attachment medium (PromoCell GmbH, Heidelberg, Germany) for 1.5 h at 37°C, non-adherent cells were aspirated and preserved in liquid nitrogen in FBS (Sigma-Aldrich, St. Louis, MO) containing 10% DMSO, and adherent monocyte cells were washed and incubated for 6 days in DC generation medium (PromoCell) with GM-CSF and IL-4, and then incubated for 48 h in the maturation medium containing TNF-α, IL-1β, IL-6 and PGE2. The resultant mature moDC (CD14low/CD45+/CD83+) were confirmed by flow cytometry. Allogenic naïve CD4 T cells were isolated the day before co-culture with moDC (see “*In vitro* model” below) using magnetic bead kit (STEMCELL Technologies, Inc., Vancouver, Canada) for isolation of untouched naïve CD4 T cells according to the manufacturer protocol. Briefly, frozen non-adherent fraction of PBMC/CBMC was thawed, cells were washed with complete RPMI (Gibco) with 10% FBS and PBS with 2% FBS and 1mM EDTA. Cells were, re-suspended in PBS with 2% FBS and 1mM EDTA, counted and incubated with appropriate amount of antibody cocktail and magnetic beads targeted against cells expressing CD8, CD14, CD16, CD19, CD20, CD25, CD36, CD45RO, CD56, CD61, CD66b, CD123, HLA-DR, TCRγ/δ, and glycophorin A surface markers. Untouched naive CD4 T cell were washed and re-suspended in complete RPMI with 10% FBS and human recombinant IL-2 at 20 ng/ml (R&D Systems, Inc., Minneapolis, MN).

### In vitro model—moDC stimulation and T cell polarization

Mature moDC prepared as described above were stimulated for 48 h with live RSV strains, Mock, FI-RSV, FI-Mock, Gwt-VLP and Gmut-VLP. The amount of virus or VLP was chosen to contain 5 μg/ml final concentration of G protein. This corresponds to ~1.0 MOI (multiplicity of infection) of live RSV which was used in earlier studies [28]. Mock and FI-mock were diluted at the same manner as live or FI-RSV for the stimulation studies. After the stimulation, supernatants were collected and stored at -80 with protease inhibitor for cytokine assay, moDC were washed and counted, 5x10^4^ cells were taken for T cell polarization assay, the rest of moDC were stained for flow cytometry with Zombie Violet (dead cell exclusion, Biolegend, San Diego, CA), anti-CD14 (monocyte exclusion, Biolegend), anti-CD83 (immature cell exclusion, Biolegend), anti-HLA-DR (BD Biosciences, San Jose, CA), anti-OX40L (BD Biosciences), anti-Jagged-1 (Biolegend).

For the T cell polarization assay, moDC were treated for 2 h with 10 μg/ml of mitomycin C and washed, and mixed at 1:4 ratio with allogenic naïve CD4 T cells. Allogenic naïve CD4 T cells were isolated and stored as described above, cells were thawed the night before co-culture with moDC and left overnight in complete RPMI at 37C to rest. Next day T cells were counted and plated at 20 x10^4^ cell per well for co-culture with allogenic moDC. MoDC and T cells were co-cultured in complete RPMI supplemented with 20 ng/ml of recombinant human IL-2 for 6 days. On day 6 of incubation supernatants were collected and stored at -80 with protease inhibitor for cytokine assay. T cells were stained for flow cytometry with Zombie Violet (dead cell exclusion, Biolegend), anti-CD4 (Biolegend), then fixed with Transcription Factor Fixation/Permeabilization solution (eBioscience, Inc., San Diego, CA), permeabilized with Permeabilization Buffer (eBioscience) and stained with anti-T-bet (Biolgend), anti-Gata-3 (eBioscience), anti-ROR-γt (Biolegend) and anti-Foxp3 (Biolegend) antibody. Flow cytometry analysis was performed using 4-laser BD LSR-II (BD Biosciences) and FlowJo software (Tree Star, Inc., Ashland, OR).

MoDC and T cell cytokines were detected in freshly thawed supernatants using Luminex magnetic bead kits (Invitrogen, Carlsbad, CA) according to the manufacturer’s instructions and analyzed on Luminex LX100 analyzer (Luminex Corporation, Austin, TX) with Bio-Plex Manager software (Bio-Rad Laboratories, Hercules, CA).

### Data analysis

Statistical analysis and graphs were performed using GraphPad Prizm software (GraphPad Software, Inc., La Jolla, CA). Data are expressed as mean ± standard error of mean (SEM) of independent experiments with PBMC/CBMC from 10–11 random donors and 3 replicates for each experimental condition. To account for variation among donors and focus on relative differences among FI-RSV, live RSVs, and VLPs, we used fold changes over background (mock) or difference, i.e. background (mock) value subtracted. The paired t-test and one-way ANOVA were employed to determine the significance of differences between experimental conditions, a P-value <0.05 was considered to be statistically significant.

### Ethics statement

Delinked peripheral blood was collected under the “Phlebotomy of Health Adults” protocol to collect blood from healthy adult donors. The protocol (IRB00045690) was reviewed and approved by the Emory University Institutional Review Board, Research Administration.

Delinked cord blood from delivery of healthy full term infants was collected under the “Placental HIV-1 and CMV Transmission Study” protocol to collect placental tissue and cord blood specimens. The protocol (IRB00021715) was reviewed and approved by the Emory University Institutional Review Board, Research Administration. Cord blood was collected from HIV-1 and CMV negative subjects.

## Results

### Assessment of type-1/type-2 directing functional phenotype in human moDC

**Fig 1:**
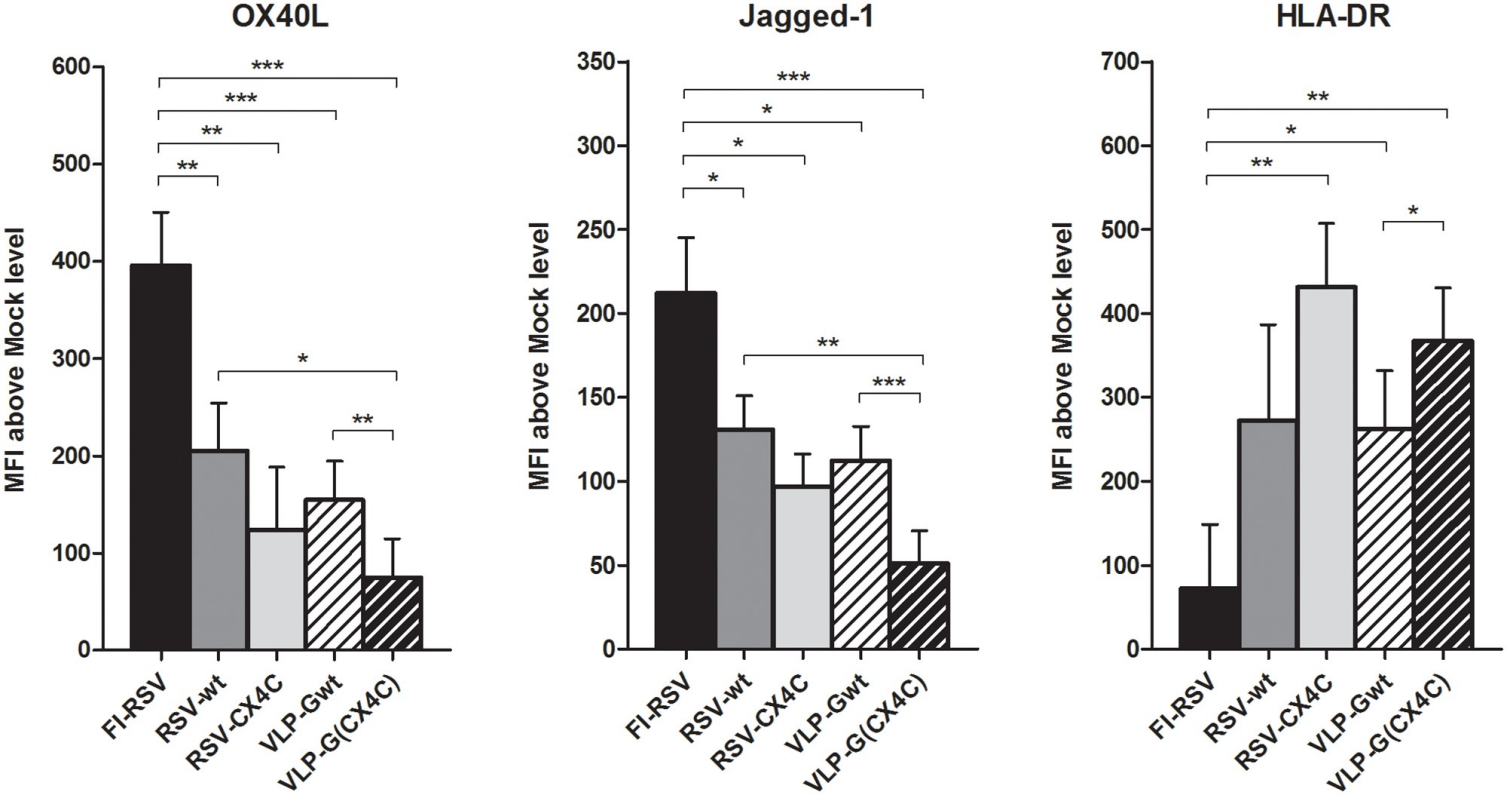
Surface marker expression on human moDC stimulated *in vitro* with live RSV, FI-RSV and VLP. Human moDC from 11 donors were stimulated for 48h with FI-RSV, FI-mock, live RSV strains A2 (RSV-wt) and A2 with mutated CX3C motif in G protein (RSV-CX4C), Mock, and VLPs containing wild-type or mutated G protein (VLP-Gwt and VLP-G(CX4C)). Data presented as Mean + SEM of mean fluorescence intensity (MFI) above background level (FI-mock and Mock respectively); *—p < 0.05, ** p < 0.01, ***—p < 0.001 by unpaired t-test.

**Fig 2:**
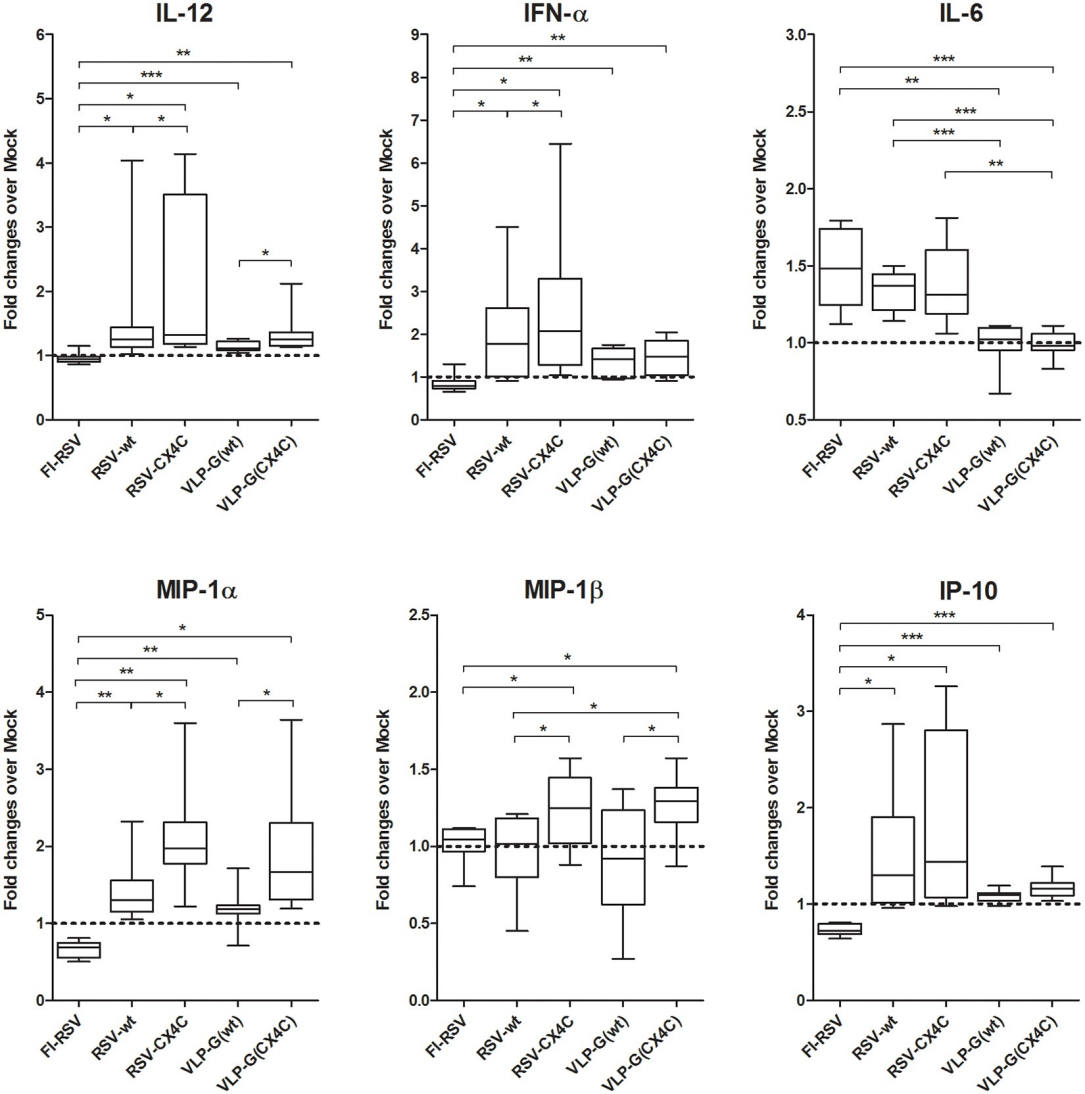
Cytokine production by human moDC stimulated *in vitro* with live RSV, FI-RSV and VLP. Human moDC were stimulated for 48h with FI-RSV, FI-mock, live RSV strains A2 (RSV-wt) and A2 with mutated CX3C motif in G protein (RSV-CX4C), Mock, and VLPs containing wild-type or mutated G protein (VLP-Gwt and VLP-G(CX4C)). Data presented as Median with range and 25–75 percentile of fold changes over background level (FI-mock and Mock respectively). Data presented from 11 PBMC/CBMC donors for IL-12, and 8 PBMC/CBMC donor for other cytokines. *—p < 0.05, ** p < 0.01, ***—p < 0.001 by unpaired t-test.

Innate immune responses are the first line of the defense against a pathogen. They also guide the development of immune memory responses including Th1, Th2, and Th17 biased responses. Dendritic cells participate in guiding memory responses and surface molecules indicate the bias of the T-cell response they direct. For example, OX40L and Jagged-1 indicate polarizing T cells toward a type-2 memory responses [31–37]. With our *in vitro* human moDC model, we determined the dendritic cell type-1/type-2 functional phenotype induced by stimulation with FI-RSV (response associated with ERD), two live RSV strains (response not associated with ERD), and an example of a subunit candidate vaccine–RSV G protein VLPs. We tested moDCs from 5 adult peripheral blood monocyte cell (PBMC) and 5 cord blood monocyte cell (CBMC) specimens. Due to fairly high subject to subject variations in individual responses, data were analyzed and presented below as values above or folds over background (mock stimulation). Two types of G-VLPs were used: VLP constructs with wild-type G (VLP-Gwt), and G with a mutation in the CX3C motif (VLP-G(CX4C)). Corresponding wild-type and mutated G live RSV A2 strains were included: RSV-wt and RSV-CX4C. Expression of moDC markers (Fig 1 and S2 Fig) and intracellular cytokines (Fig 2 and S1A Table) demonstrate that, as suggested by earlier studies, FI-RSV directs responses towards type-2 while live RSV towards more type-1 biased response. We found a significantly higher expression of OX40L and Jagged-1 for FI-RSV compared to RSV-wt, RSV-CX4C, VLP-Gwt and VLP-G(CX4C) (Fig 1). Although moDC derived from PBMC compared to those derived from CBMC showed quantitative differences, e.g. the decrease in HLA-DR expression was more pronounced in CBMC-derived moDCs, the qualitative relationship among responses to FI-RSV, the two live RSV strains, and the two VLPs were similar (S3 and S4 Figs). The lower HLA-DR expression seen after FI-RSV stimulation suggests it dampened dendritic cell activation. Importantly, both G VLPs induced levels of OX40L and Jagged-1 similar to live RSV, i.e. indicative of a type-1 guiding response and not the FI-RSV-associated type-2 biased response.

The cytokine microenvironment plays an important role in directing immune responses, with higher levels of DC-produced IL-12 associated with a Th1 differentiation from naïve CD4 T cell while higher levels of IL-6 promote Gata3 upregulation and Th2 biased T cell response [38–41]. Our results for moDC cytokine production also suggest a type-2 biased response after FI-RSV stimulation and a type-1 biased response after stimulation with the two live viruses and the two VLPs (Fig 2). Note the lack of an increase in IL-12 over background for FI-RSV and significant increase for the viruses and VLPs. In contrast, FI-RSV-stimulated moDCs produced higher levels of IL-6 than cells stimulated with live viruses or VLPs. These differences were statistically significant only for VLPs. Of interest, the two live viruses and two VLPs also induced significantly higher levels of IFN-α, MIP-1α and IP-10 in moDC. The decreased levels of these cytokines after FI-RSV stimulation suggest an overall lower quality of the immune response induced by this vaccine. IFN-α is an important anti-viral agent [42, 43] and also involved in primary CD8 T cell responses [44]. Together with OX40L, it is crucial for the induction of primary CD4 T cell responses [45]. MIP-1α/1β and IP-10 are T cell chemo-attractants and play important role in the development of protective Th1 and CD8 T cell responses [46–48]. These results demonstrate distinct differences in cytokines induced by FI-RSV and live RSV and also show that the response to these VLPs are significantly different from the FI-RSV response and qualitatively similar to live RSV responses. This suggests these VLPs may not be a risk for ERD-associated immunopathology.

We were also interested in the effect of blocking G binding to CX3CR1 on moDC responses. In earlier studies, we have noted differences in dendritic and/or T cell responses between stimulation with RSV-wt and RSV-CX4C both in *in vitro* with human PBMCs and during the RSV infection in mice [25, 28]. In the present study, consistent with the earlier studies, G with the CX4C mutation in either live virus or VLPs compared to wildtype G was associated with moDC responses that direct a more type-1 versus type-2 type response. For example for the live viruses, G with the CX4C mutation was associated with higher levels of IL-12, IFN-α, MIP-1α, and MIP-1β (Fig 2). For the VLPs, the G with the CX4C mutation compared to wildtype G induced significantly lower expression of OX40L and Jagged-1, higher expression of HLA-DR (Fig 1), and higher levels of IL-12, MIP-1α and MIP-1β in moDC (Fig 2). These data support our earlier findings with the two forms of G and suggest that preventing the G-CX3CR1 interaction may induce safer immune responses.

### Allogenic T cell responses induced by type-1/type-2 phenotype moDC

To validate and extend our findings of differential moDC functional phenotype induced by FI-RSV vs live viruses or VLPs, we evaluated T cells responses promoted by these moDC. In these experiments, we co-cultured human naïve CD4 T cells with their allogenic previously stimulated moDC *in vitro* as noted above. The stimulated moDC were treated with mitomycin C before co-culture to avoid further proliferation while preserving antigen-presentation and co-stimulatory signaling.

**Fig 3:**
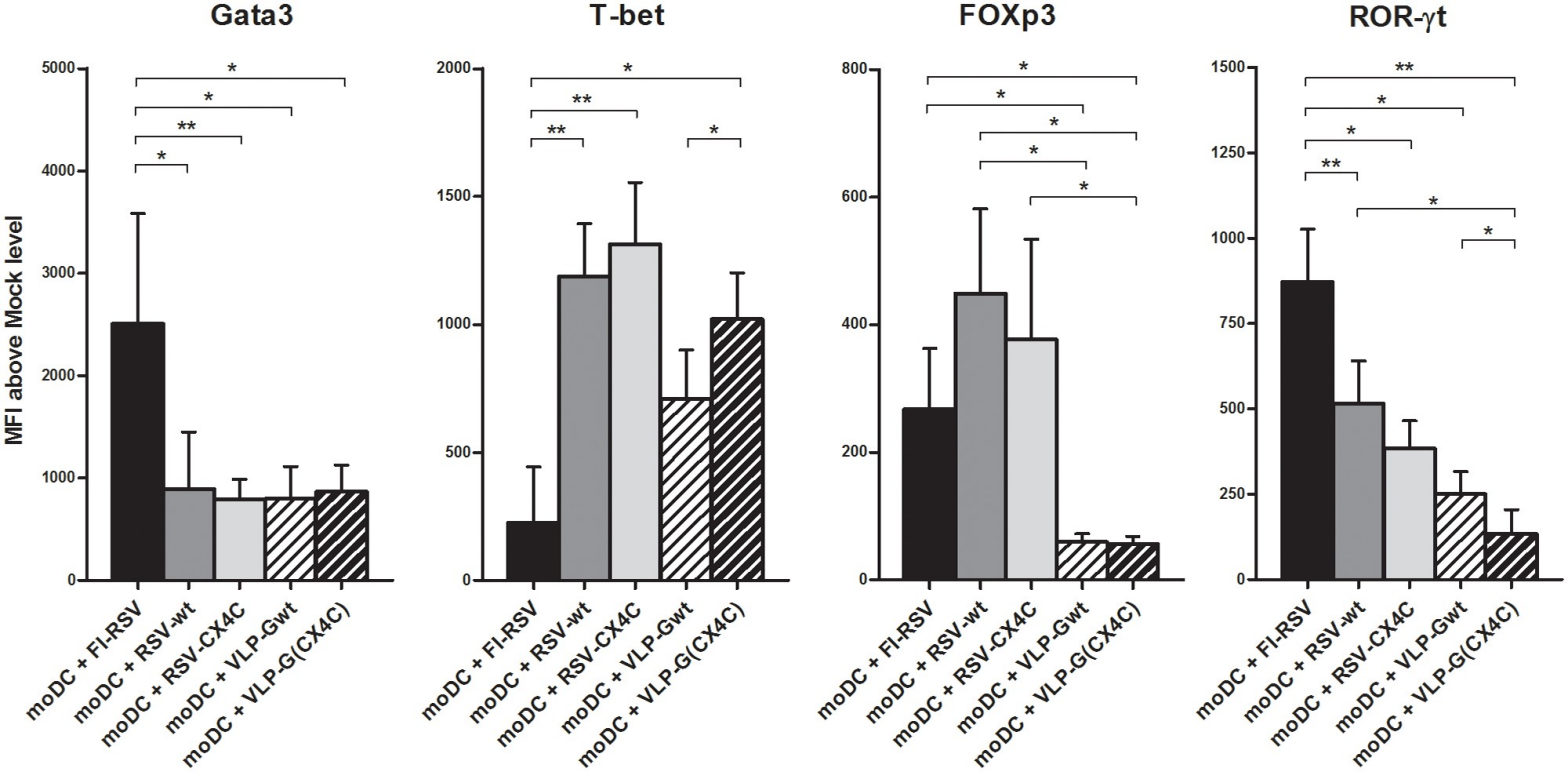
Transcription factor expression in human CD4 T cell after co-culture with allogenic moDC stimulated with live RSV, FI-RSV and VLP. Human naïve CD4 T cells from 10 donors were co-cultured for 4 days with allogenic moDC previously stimulated *in vitro* with FI-RSV, FI-mock, live RSV strains A2 (RSV-wt) and A2 with mutated CX3C motif in G protein (RSV-CX4C), Mock, and VLPs containing wild-type or mutated G protein (VLP-Gwt and VLP-G(CX4C)). Data presented as Mean + SEM of mean fluorescence intensity (MFI) above background level (FI-mock and Mock respectively). *—p < 0.05, ** p < 0.01, ***—p < 0.001 by unpaired t-test.

Live RSV-stimulated moDCs compared to mock stimulated moDCs induced predominantly T-bet responses in allogenic T cells plussome increases in expression of other transcriptional factors (Fig 3 and S5 Fig). Similar to live strains, VLP-stimulated moDC induced high expression of T-bet, while FI-RSV-stimulated moDC resulted in significantly elevated Gata3 and ROR-γt expression with relatively low T-bet expression in allogenic T cells. These findings suggest that FI-RSV stimulated moDC direct CD4 T cell differentiation predominantly towards Th2/Th17. We did not see significant differences in Foxp3 expression between FI-RSV and live RSV strains, while moDC stimulated with G-VLP induced significantly lower expression of Foxp3 in CD4 T cells.

**Fig 4:**
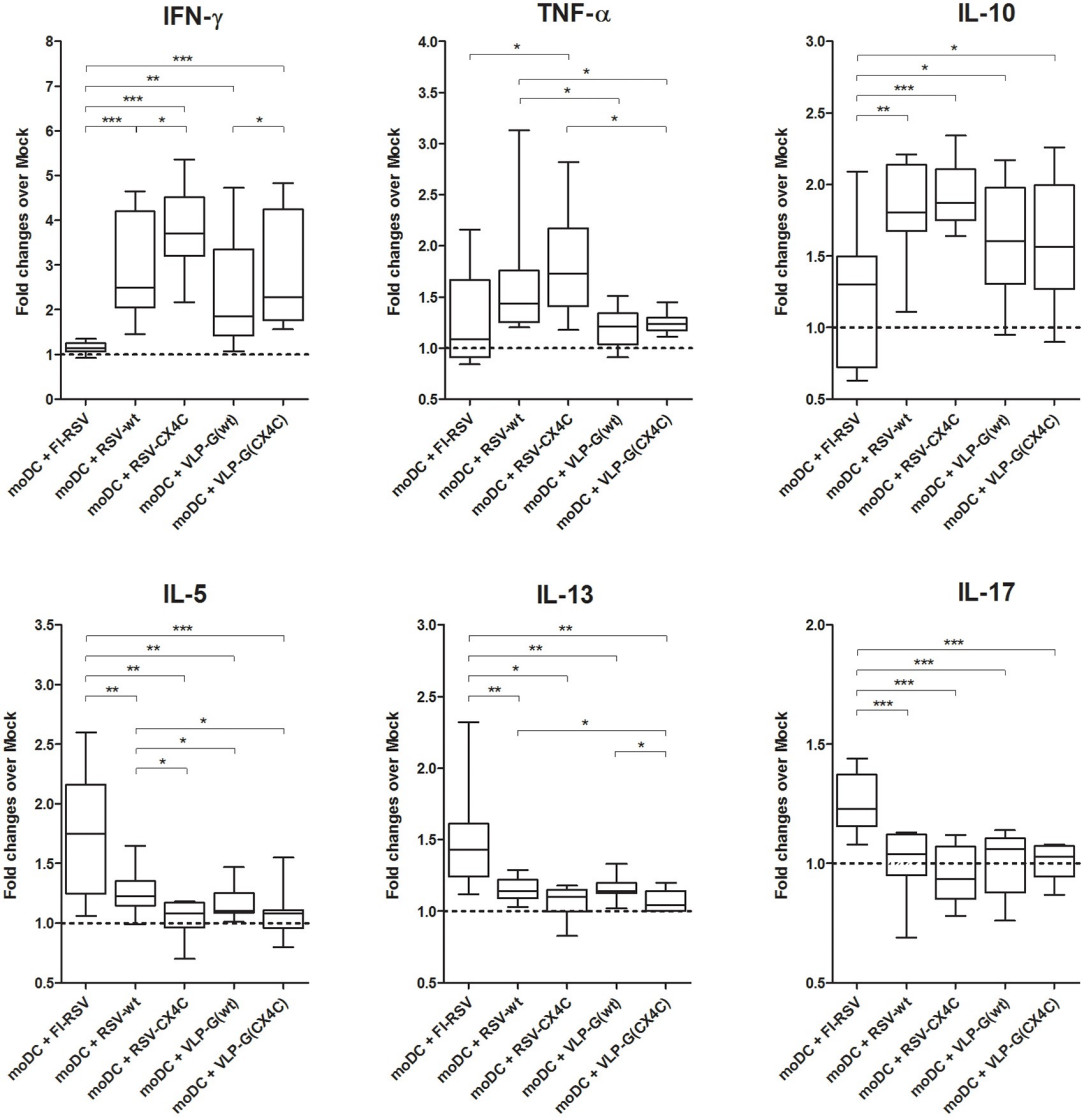
Cytokine production by human CD4 T cell after co-culture with allogenic moDC stimulated with live RSV, FI-RSV and VLP. Human naïve CD4 T cells from 10 donors were co-cultured for 4 days with allogenic moDC previously stimulated *in vitro* with FI-RSV, FI-mock, live RSV strains A2 (RSV-wt) and A2 with mutated CX3C motif in G protein (RSV-CX4C), Mock, and VLPs containing wild-type or mutated G protein (VLP-Gwt and VLP-G(CX4C)). Data presented as Median with range and 25–75 percentile of fold changes over background level (FI-mock and Mock respectively). *—p < 0.05, ** p < 0.01, ***—p < 0.001 by unpaired t-test.

Analysis of cytokines produced by moDC-stimulated T cells confirmed the transcriptional factor data (Fig 4 and S1B Table). FI-RSV-stimulated moDC induced high levels of IL-5/IL-13 in T cell cultures and low levels of IFN-γ/TNF-α, as compared to live RSV and G-VLP constructs. In addition, FI-RSV stimulated moDC induced limited production of IL-17. Although responses were overall low for all samples, the differences between FI-RSV and live RSV and G-VLPs were consistent among all donors and were significant. These data largely correspond to the differential functional phenotype of stimulated moDC observed above. Live RSV induced a more balanced type-1/type-2 DC phenotype and the corresponding T cell differentiation, while FI-RSV induced responses skewed toward Th2/Th17 phenotype. In addition, FI-RSV stimulated moDC induced significantly lower production of IL-10 in CD4 T cells compared to live RSV- and VLP-stimulated moDC. Since FI-RSV stimulated moDC induced expression of Foxp3 similar to live RSV, the lower levels of IL-10 suggest FI-RSV stimulated Tregs may have altered functionality.

Mutation of CX3C site in RSV G protein increased type-1 T cell responses, and these differences were most clear in moDC stimulated with G-VLPs. T cells co-cultured with VLP-G(CX4C)-stimulated moDC had higher levels of T-bet and IFN-γ expression, and lower of ROR-γt and IL-13, as compared to VLP-Gwt (Figs 3 and 4). Differences between live RSV strains were noted only in T cell cytokine levels. RSV-CX4C-stimulated moDC induced higher production of IFN-γ and lower levels of IL-5 in the co-cultured allogenic T cells. These data suggest that CX3C motif in RSV G protein participates in inducing type-2 directing moDC, likely due to its ability to bind to CX3CR1 expressed on immune cells, including dendritic cells [49–51].

## Discussion

The search for a vaccine has been a goal in RSV research for more than 50 years. The failure of the first RSV vaccine, FI-RSV, that induced ERD in vaccinated children with later natural RSV infection, set a major roadblock in the vaccine development path. Concern about the safety of the vaccine candidates in regard to ERD has so far precluded development of subunit non-live virus vaccines for young children. Though animal models including mice, cotton rats, lambs, calves and non-human primates provide important information on pathogenesis of ERD and are used to assess a candidate vaccine’s risk of predisposing to ERD [52–57], information on the human ERD-associated immune response is needed to determine the ERD-risk of a non-live virus vaccine. The inconsistency between animal species and different studies in the same species further complicates applying animal model datato humans. The only data on the human response associated with FI-RSV comes from the original trials when limited immunological tools did not include measures of the biomarkers subsequently linked to FI-RSV ERD. A number of recent *in vitro* studies have looked at various human immune responses to RSV in PBMC [58–68] but only one, Jackson et al., compared human responses to live RSV and FI-RSV [61]. In that study, however, they assessed memory T cell associated responses in RSV-primed adult PBMCs. They found a type-1 T cell responses after live RSV stimulation and Th2-skewed responses after FI-RSV stimulation. In the current paper, we describe a human *in vitro* model of immune responses likely to be induced by a vaccine rather than those based on prior memory. One caveat in our in vitro assessment of the FI-RSV is that the preparation used was not shown to induce enhanced disease in animals. However, we have shown similar FI-RSV preparations caused enhanced disease in animals and the responses induced in our *in vitro* model are consistent with the Th2 biased response in animals associated with enhanced disease. This model identifies multiple differences between live RSV and FI-RSV induced moDC responses associated with directing T cell differentiation. We hypothesize that these differences can be used to assess a vaccine’s risk of predisposing to enhanced.

The importance of monocytes and monocytic cell lines in RSV infection and ERD immunopathogenesis was shown previously in various animal models and human *in vitro* studies including promotion and polarization of the responses to RSV [57, 69–75]. Monocyte/macrophage pulmonary infiltration was also observed by Prince et al. during their re-examination of autopsy specimen from the original failed FI-RSV trial [76]. Monocytic IL-12 production has also inversely correlated with the severity of infection in infants [70]. Our data showed that FI-RSV induces type-2 polarizing moDC phenotype with high expression of OX40L and Jagged-1, and subsequently Th2 differentiation in allogenic T cells. Live RSV strains and G-VLP constructs, although also increased OX40L and Jagged-1 expression compared to mock but to lower levels than FI-RSV and induced other responses indicative of type-1 T cell responses. The decreased level of type-2 responses to live virus and VLPs was associated with elevated production of IL-12 and IFN-α in moDC. It has been noted that Th2-polarizing ability of OX40L is significantly decreased in the presence of IL-12 [77]. Additionally, recent studies show that OX40L and Jagged-1 participate in dendritic cell maturation and antigen presentation [78–80], suggesting their importance for both type-2 and type-1 responses. These studies and our data correspond to the findings that OX40 and Notch signaling is essential for T cell stimulation but not necessarily with a type-2 bias [81, 82]. The type-2 bias is also affected by the accompanying cytokine environment.

OX40L and Jagged-1 have also been noted to have a role in the proliferation of Tregs [83–85]. We observed a fairly high expression level of Foxp3 in T cells pulsed by FI-RSV-stimulated moDC. This increased expression of Foxp3, however, was not accompanied by production of IL-10, as opposed to live RSV strains that had both elevated Foxp3 and IL-10 levels. These data suggest that that FI-RSV may induce differentiation of non-suppressive Treg or Treg with altered functionality. While the importance of Treg in regulation and suppression of RSV-induced pulmonary inflammation has been described [86–90], there is limited data on the role of Treg in ERD. Studies have shown decreased numbers of CD4+Foxp3+ cells in lungs of FI-RSV vaccinated mice after live virus challenge [18, 91] but not altered functionality of FI-RSV-induced Treg responses as suggested by our *in vitro* data.

We developed this model as an additional pre-clinical tool to evaluate ERD-risk of a vaccine and used G-VLPs as a test case. Studies in animals have found G-VLP vaccines and G peptide vaccines to induce immune responses that do not predispose to ERD [26, 92, 93]. In fact, in an earlier study a G-peptide vaccine given with FI-RSV prevented ERD in mice later challenged with RSV [26]. Thus, our data is consistent with and supports animal studies that suggest a G or G peptide vaccine will not predispose to ERD.

We also used the model to assess the effect of blocking G-binding to CX3CR1 by including the virus and G-VLP with CX3C site mutated to CX4C. Similar to earlier *in vitro* and mouse studies, the CX4C mutation improved the quality of the immune response, i.e. the G(CX4C) compared to Gwt in live RSV and G-VLPs led to an increased production of IL-12 and MIP-1α/1β (both live virus and VLPs) and IFN-α (live virus only) in moDC that was also associated with lower expression of OX40L and Jagged-1 and increased expression of HLA-DR (VLPs only). Since cytokine microenvironment plays a crucial role in the direction of DC-T cell signaling [77], it is likely that higher production of IL-12 contributed to a more prominent type-1 response (higher T-bet and IFN-γ expression, and lower IL-5/IL-13 production) in T cell cultures pulsed by moDC stimulated with G(CX4C) virus/VLPs, as compared to the wild-type G live RSV and VLPs. These support the concept that the CX3C motif in G is immunologically active in RSV disease and modifying it can improve a G vaccine safety profile.

In summary, our *in vitro* model of human immune responses to RSV is consistent with animal model data that show the ERD-risk of FI-RSV through priming innate responses to direct T cells towards type-2. A combination of biomarkers in dendritic cells such as increased OX40L/Jagged-1 expression and decreased IL-12/IFN-α production and naïve T cells pulsed with stimulated dendritic cells such elevated Gata3 and ROR-γt expression provide robust measures of immune responses likely associated with ERD risk. This in vitro human model presents a tool to evaluate a vaccine’s risk of predisposing to ERD that should help move the field closer to developing a subunit, non-live virus vaccine for young children.

## Supporting information

S1 Raw image Westernblot of G protein expression on VLPs. Original raw image of the Western blot presented in S1A Fig. VLPs purified by 20% sucrose cushion centrifugation were resolved by SDS-PAGE and analyzed by western blot. VLP-Gwt (line 1) or VLP-G(CX4C) (line 4) were blotted by human anti-G antibody (3D3, Trellis Bioscience LLC, Redwood City, CA).
(PDF)

S1 Fig VLP evaluation. **A.** Westernblot of G protein expression on VLPs. VLPs purified by 20% sucrose cushion centrifugation were resolved by SDS-PAGE and analyzed by western blot. VLP-Gwt or VLP-G(CX4C) were blotted by human anti-G antibody (3D3, Trellis Bioscience LLC, Redwood City, CA). **B.** Electron microscopy of VLP shape and expression of G protein. Purified VLP-Gwt were labeled with human anti-G antibody (3D3, Trellis Bioscience LLC, Redwood City, CA) followed by gold-conjugated secondary antibody. Bar 50 nm. **C.** G protein titration by ELISA. Purified G protein and purified VLPs were immobilized on a 96-well plate at different concentrations in a two-fold serial dilution fashion. Plate was blocked followed by incubation with human anti-G antibody (3D3) and HRP-conjugated antibody. OPD was used as substrate to develop reaction and the absorbance read at 490 nm. The G protein amount in VLP-Gwt or VLP-G(CX4C) was calculated based on the standard curve.
(TIF)

S2 Fig Surface marker expression on human moDC stimulated *in vitro* with live RSV, FI-RSV and VLP. Human moDC were stimulated for 48h with FI-RSV, live RSV strains A2 (RSV-wt) and A2 with mutated CX3C motif in G protein (RSV-CX4C), and VLPs containing wild-type or mutated G protein (VLP-Gwt and VLP-G(CX4C)). Representative histograms show marker expression in mock (grey background), FI-RSV, RSV-wt, VLP-Gwt (solid line), and RSV-CX4C, VLP-G(CX4C) (dotted line).
(TIF)

S3 Fig Surface marker expression on human PBMC- and CBMC-derived moDC stimulated *in vitro* with live RSV, FI-RSV and VLP. Human moDC from 6 PBMC and 5 CBMC donors were stimulated for 48h with FI-RSV, FI-mock, live RSV strains A2 (RSV-wt) and A2 with mutated CX3C motif in G protein (RSV-CX4C), Mock, and VLPs containing wild-type or mutated G protein (VLP-Gwt and VLP-G(CX4C)). Data presented as Mean + SEM of mean fluorescence intensity (MFI) above background level (FI-mock and Mock respectively); *—p < 0.05, ** p < 0.01, ***—p < 0.001 by unpaired t-test.
(TIF)

S4 Fig Cytokine production by human PBMC- and CBMC-derived moDC stimulated *in vitro* with live RSV, FI-RSV and VLP. Human moDC were stimulated for 48h with FI-RSV, FI-mock, live RSV strains A2 (RSV-wt) and A2 with mutated CX3C motif in G protein (RSV-CX4C), Mock, and VLPs containing wild-type or mutated G protein (VLP-Gwt and VLP-G(CX4C)). Data presented as Median with range and 25–75 percentile of fold changes over background level (FI-mock and Mock respectively). Data presented from 6 PBMC and 5 CBMC donors for IL-12, and 4 PBMC and 4 CBMC donors for other cytokines. *—p < 0.05, ** p < 0.01, ***—p < 0.001 by unpaired t-test.
(TIF)

S5 Fig Transcription factor expression in human CD4 T cells after co-culture with allogenic moDC stimulated with live RSV, FI-RSV and VLP. Human naïve CD4 T cells were co-cultured for 4 days with allogenic moDC previously stimulated *in vitro* with FI-RSV, FI-mock, live RSV strains A2 (RSV-wt) and A2 with mutated CX3C motif in G protein (RSV-CX4C), Mock, and VLPs containing wild-type or mutated G protein (VLP-Gwt and VLP-G(CX4C)). Representative histograms show marker expression in mock (grey background), FI-RSV, RSV-wt, VLP-Gwt (solid line), and RSV-CX4C, VLP-G(CX4C) (dotted line).
(TIF)

S1 Table **A.** Cytokine production by human moDC stimulated *in vitro* with live RSV, FI-RSV and VLP. **B.** Cytokine production by human CD4 T cell after co-culture with allogenic moDC stimulated with live RSV, FI-RSV and VLP.
(PDF)

S1 Data Data presented in the manuscript are available in the supporting information excel file organized according to figures. (XLSX)
