# Supplementary figures and images for: In vitro model for the assessment of human immune responses to subunit RSV vaccines
Source: PLoS One. 2020 Mar 19;15(3):e0229660. doi: 10.1371/journal.pone.0229660 (PMC7081972; doi:10.1371/journal.pone.0229660)

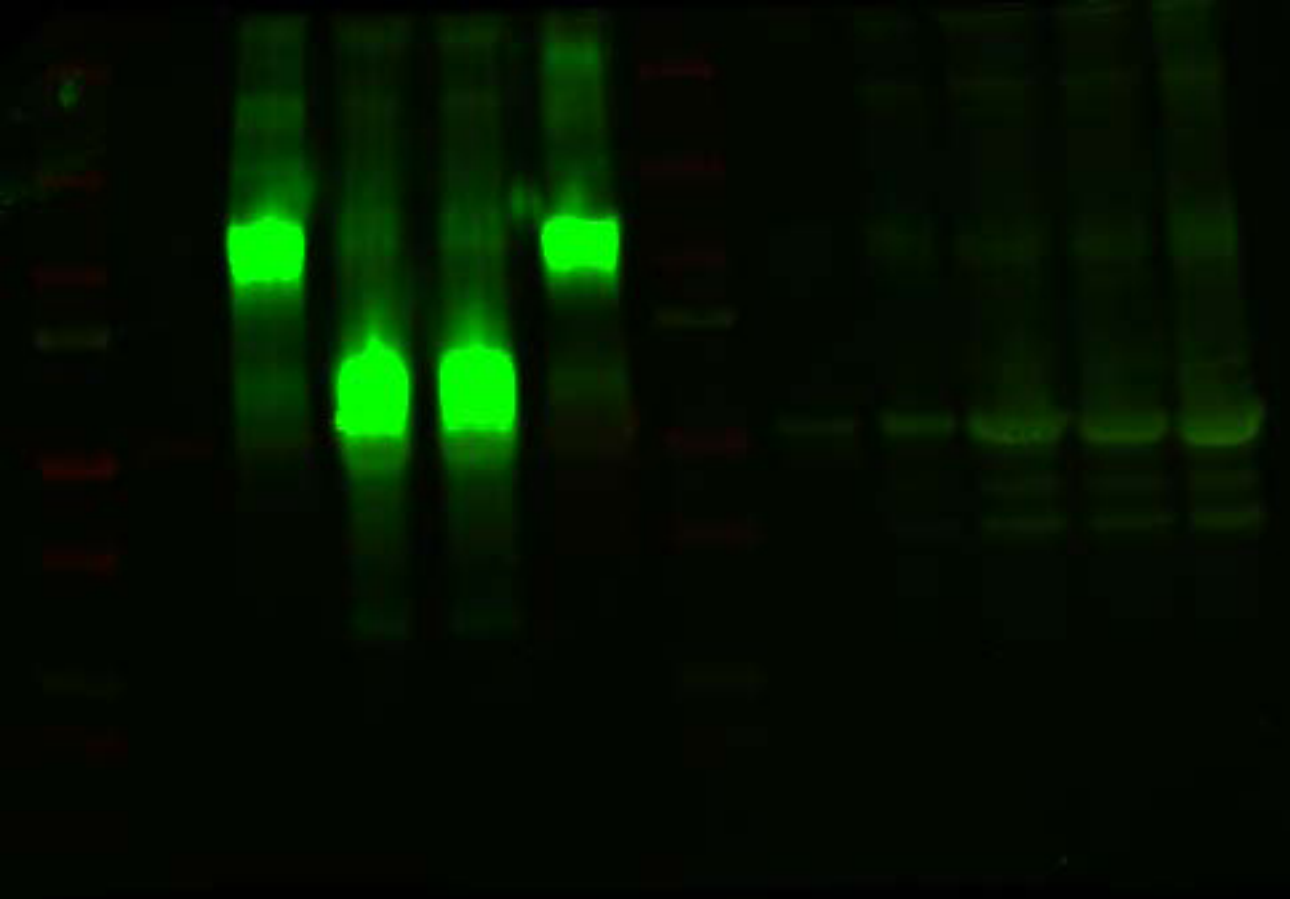

Supplement: S1 Raw image — Original raw image of the Western blot presented in S1A Fig. VLPs purified by 20% sucrose cushion centrifugation were resolved by SDS-PAGE and analyzed by western blot. VLP-Gwt (line 1) or VLP-G(CX4C) (line 4) were blotted by human anti-G antibody (3D3, Trellis Bioscience LLC, Redwood City, CA). (PDF) [file pone.0229660.s001.pdf]

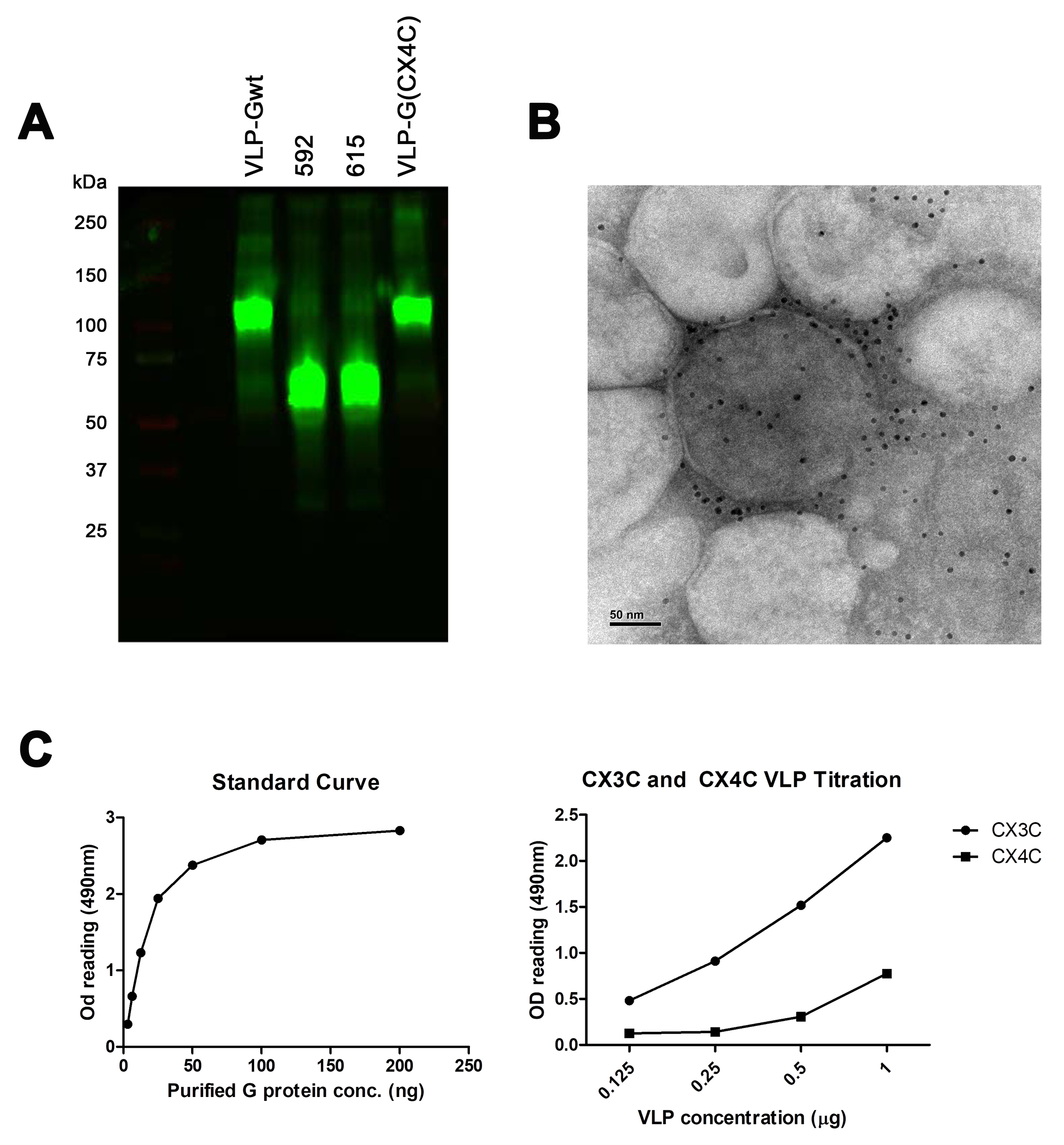

Supplement: S1 Fig — A. Westernblot of G protein expression on VLPs. VLPs purified by 20% sucrose cushion centrifugation were resolved by SDS-PAGE and analyzed by western blot. VLP-Gwt or VLP-G(CX4C) were blotted by human anti-G antibody (3D3, Trellis Bioscience LLC, Redwood City, CA). B. Electron microscopy of VLP shape and expression of G protein. Purified VLP-Gwt were labeled with human anti-G antibody (3D3, Trellis Bioscience LLC, Redwood City, CA) followed by gold-conjugated secondary antibody. Bar 50 nm. C. G protein titration by ELISA. Purified G protein and purified VLPs were immobilized on a 96-well plate at different concentrations in a two-fold serial dilution fashion. Plate was blocked followed by incubation with human anti-G antibody (3D3) and HRP-conjugated antibody. OPD was used as substrate to develop reaction and the absorbance read at 490 nm. The G protein amount in VLP-Gwt or VLP-G(CX4C) was calculated based on the standard curve. (TIF) [file pone.0229660.s002.tif]

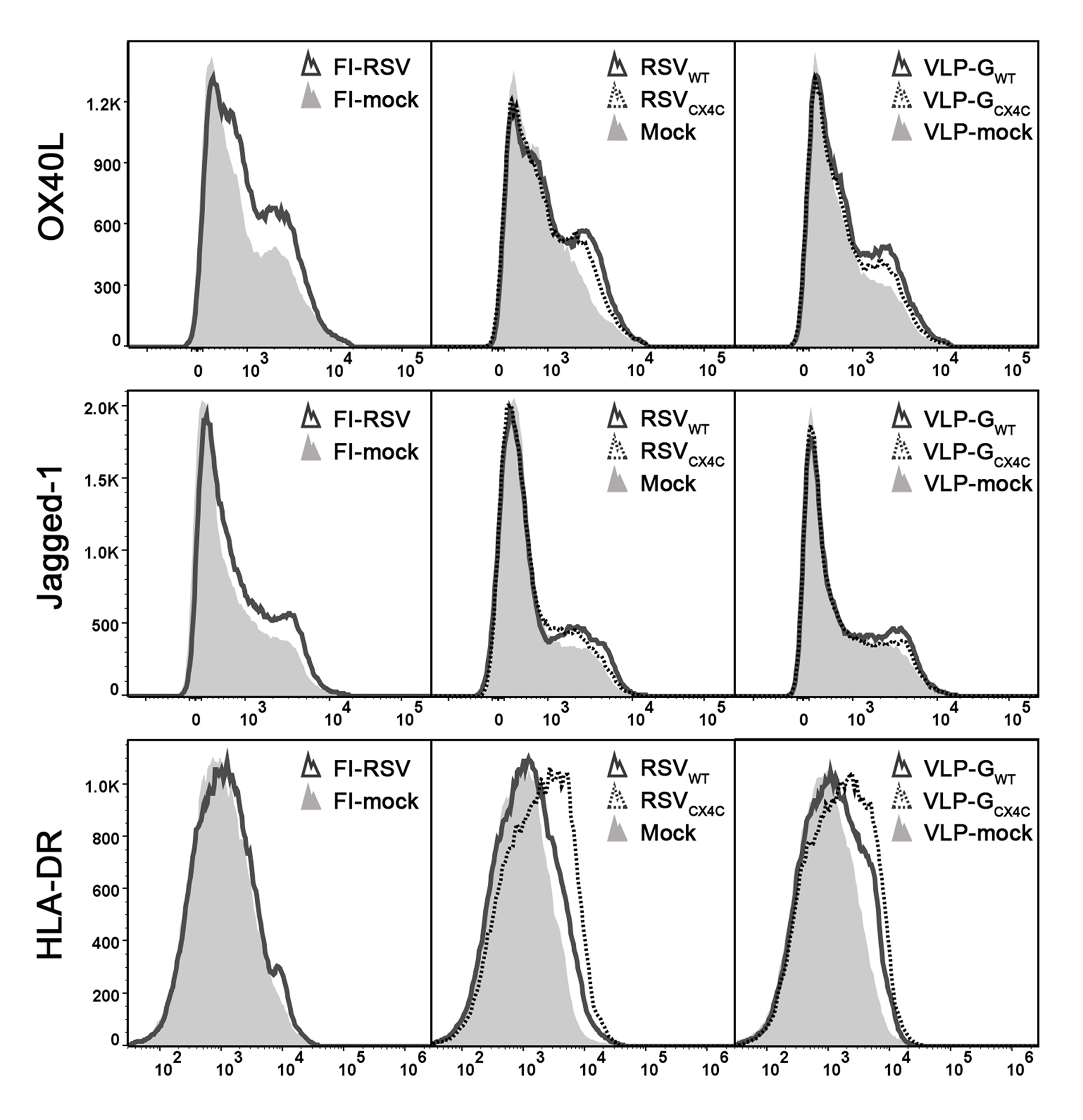

Supplement: S2 Fig — Human moDC were stimulated for 48h with FI-RSV, live RSV strains A2 (RSV-wt) and A2 with mutated CX3C motif in G protein (RSV-CX4C), and VLPs containing wild-type or mutated G protein (VLP-Gwt and VLP-G(CX4C)). Representative histograms show marker expression in mock (grey background), FI-RSV, RSV-wt, VLP-Gwt (solid line), and RSV-CX4C, VLP-G(CX4C) (dotted line). (TIF) [file pone.0229660.s003.tif]

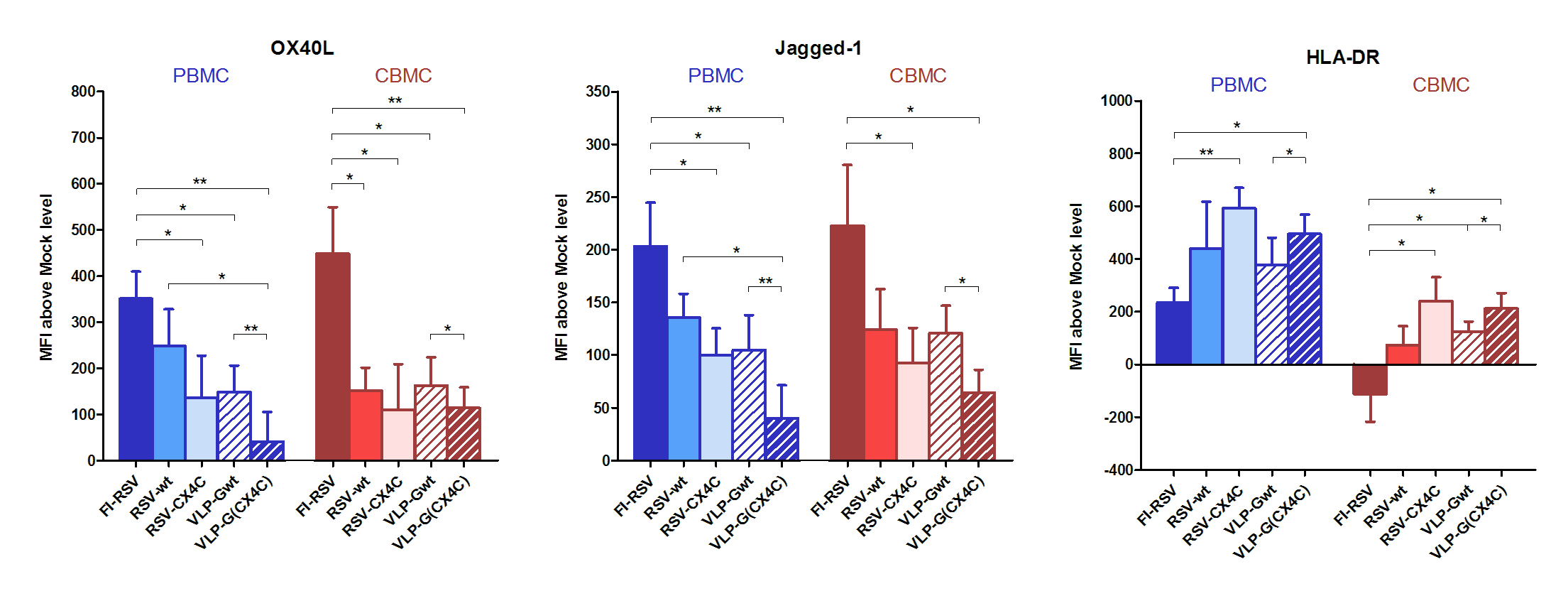

Supplement: S3 Fig — Human moDC from 6 PBMC and 5 CBMC donors were stimulated for 48h with FI-RSV, FI-mock, live RSV strains A2 (RSV-wt) and A2 with mutated CX3C motif in G protein (RSV-CX4C), Mock, and VLPs containing wild-type or mutated G protein (VLP-Gwt and VLP-G(CX4C)). Data presented as Mean + SEM of mean fluorescence intensity (MFI) above background level (FI-mock and Mock respectively); *—p < 0.05, ** p < 0.01, ***—p < 0.001 by unpaired t-test. (TIF) [file pone.0229660.s004.tif]

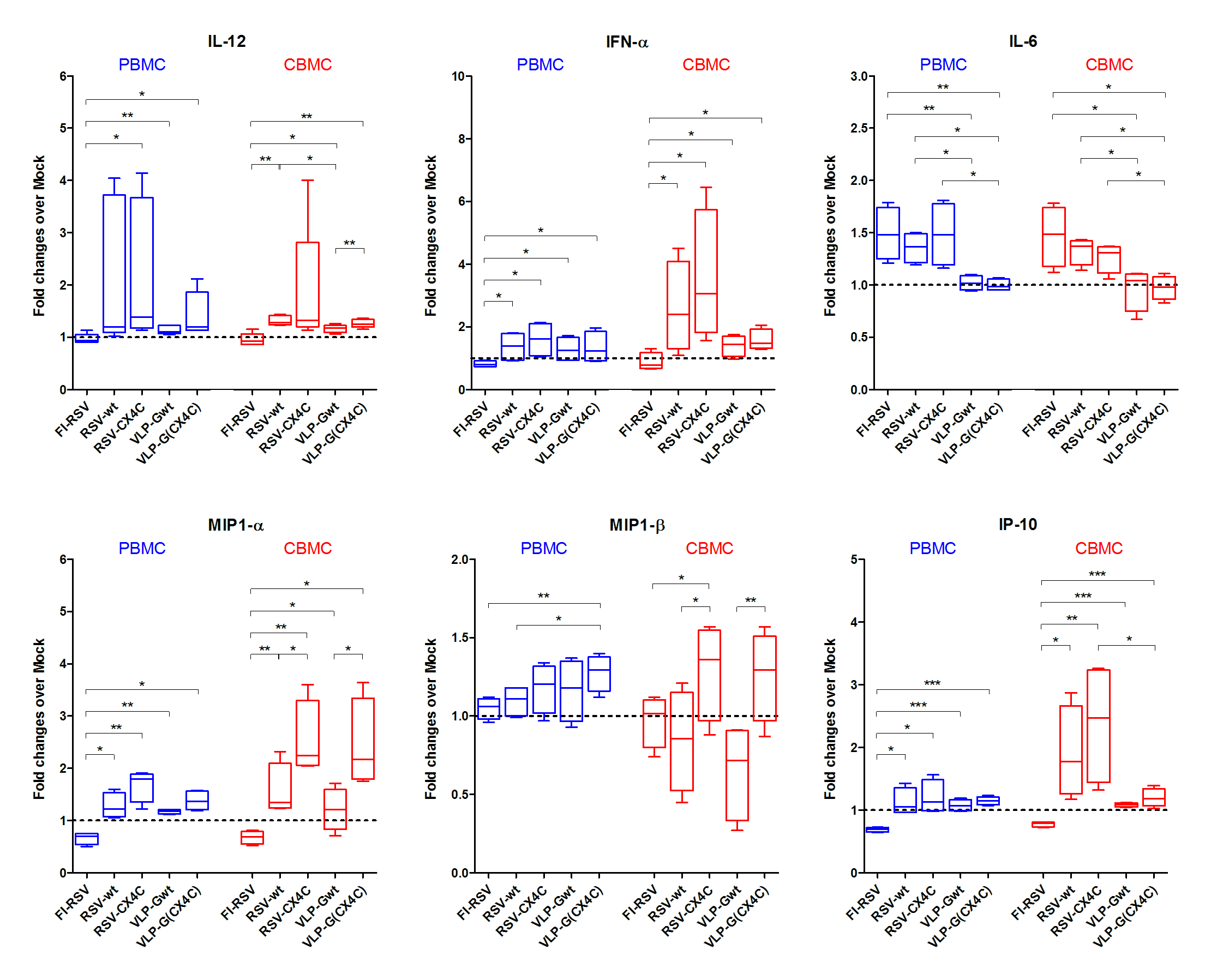

Supplement: S4 Fig — Human moDC were stimulated for 48h with FI-RSV, FI-mock, live RSV strains A2 (RSV-wt) and A2 with mutated CX3C motif in G protein (RSV-CX4C), Mock, and VLPs containing wild-type or mutated G protein (VLP-Gwt and VLP-G(CX4C)). Data presented as Median with range and 25–75 percentile of fold changes over background level (FI-mock and Mock respectively). Data presented from 6 PBMC and 5 CBMC donors for IL-12, and 4 PBMC and 4 CBMC donors for other cytokines. *—p < 0.05, ** p < 0.01, ***—p < 0.001 by unpaired t-test. (TIF) [file pone.0229660.s005.tif]

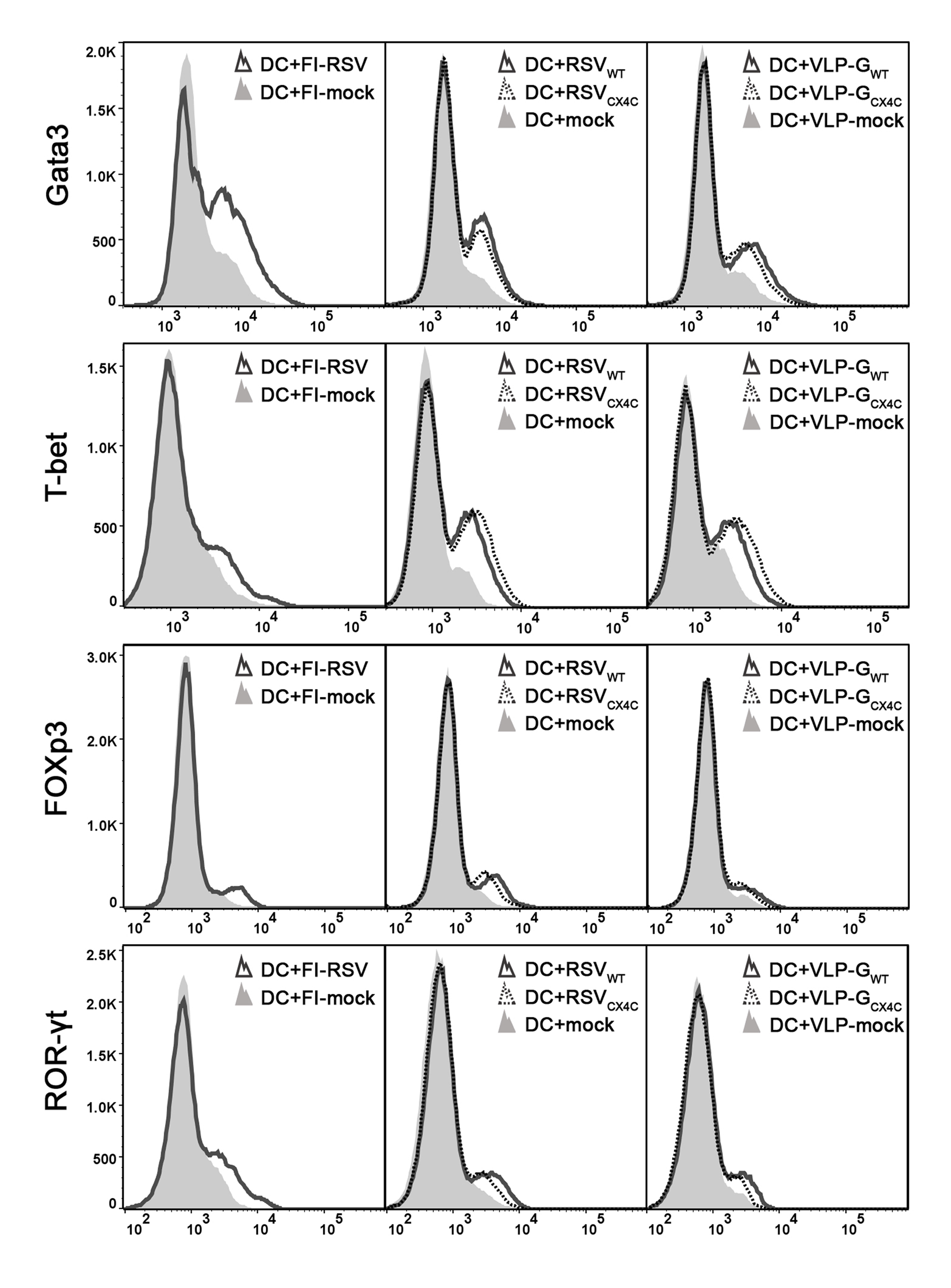

Supplement: S5 Fig — Human naïve CD4 T cells were co-cultured for 4 days with allogenic moDC previously stimulated in vitro with FI-RSV, FI-mock, live RSV strains A2 (RSV-wt) and A2 with mutated CX3C motif in G protein (RSV-CX4C), Mock, and VLPs containing wild-type or mutated G protein (VLP-Gwt and VLP-G(CX4C)). Representative histograms show marker expression in mock (grey background), FI-RSV, RSV-wt, VLP-Gwt (solid line), and RSV-CX4C, VLP-G(CX4C) (dotted line). (TIF) [file pone.0229660.s006.tif]
