# Supplementary material for: In vitro model for the assessment of human immune responses to subunit RSV vaccines
Source: PLoS One. 2020 Mar 19;15(3):e0229660. doi: 10.1371/journal.pone.0229660 (PMC7081972; doi:10.1371/journal.pone.0229660)
Supplement: S1 Table — A. Cytokine production by human moDC stimulated in vitro with live RSV, FI-RSV and VLP. B. Cytokine production by human CD4 T cell after co-culture with allogenic moDC stimulated with live RSV, FI-RSV and VLP. (PDF) [file pone.0229660.s007.pdf]

Table A. Cytokine production by human moDC stimulated *in vitro* with live RSV, FI-RSV and VLP

	Cytokine concentration (pg/ml)					
	IL-12	IFN-α	IL-6	MIP-1α	MIP-1β	IP-10
**FI-mock**	360 ± 56	102 ± 13	6246 ± 686	3481 ± 578	433 ± 37	83 ± 13
**FI-RSV**	346 ± 66	83 ± 9	9032 ± 973	2197 ± 227	433 ± 37	60 ± 8
**Mock**	436 ± 70	143 ± 18	5285 ± 796	1664 ± 177	361 ± 35	121 ± 14
**RSV-wt**	675 ± 89	260 ± 43	6988 ± 959	2277 ± 213	338 ± 36	171 ± 20
**RSV-CX4C**	786 ± 101	336 ± 61	7014 ± 898	3296 ± 271	443 ± 44	201 ± 29
**VLP-mock**	447 ± 50	124 ± 12	5809 ± 854	3123 ± 479	412 ± 40	136 ± 16
**VLP-Gwt**	510 ± 57	163 ± 20	5837 ± 990	3845 ± 762	367 ± 64	146 ± 16
**VLP-G(CX4C)**	589 ± 57	179 ± 26	5790 ± 947	5843 ± 1066	510 ± 42	157 ± 15

Table B. Cytokine production by human CD4 T cell after co-culture with allogenic moDC stimulated with live RSV, FI-RSV and VLP.

	Cytokine concentration (pg/ml)					
	IFN-γ	IL-5	IL-13	TNF-α	IL-17	IL-10
**FI-mock**	549 ± 70	677 ± 77	2811 ± 531	2510 ± 672	265 ± 50	110 ± 16
**FI-RSV**	629 ± 110	1070 ± 282	3714 ± 668	3088 ± 818	329 ± 56	121 ± 14
**Mock**	728 ± 29	395 ± 87	2247 ± 391	1145 ± 335	230 ± 57	89 ± 11
**RSV-wt**	2084 ± 230	482 ± 60	2602 ± 451	1655 ± 411	248 ± 63	169 ± 28
**RSV-CX4C**	2738 ± 302	412 ± 55	2298 ± 386	1712 ± 410	223 ± 45	165 ± 23
**VLP-mock**	783 ± 184	556 ± 76	2296 ± 379	1156 ± 337	239 ± 50	115 ± 14
**VLP-Gwt**	1839 ± 397	630 ± 62	2672 ± 438	1418 ± 357	267 ± 61	200 ± 31
**VLP-G(CX4C)**	2170 ± 278	564 ± 86	2499 ± 410	1433 ± 346	248 ± 49	187 ± 34
